# HMGB1 contributes to glomerular endothelial cell injury in ANCA‐associated vasculitis through enhancing endothelium–neutrophil interactions

**DOI:** 10.1111/jcmm.13065

**Published:** 2017-02-09

**Authors:** Chen Wang, Dong‐Yuan Chang, Min Chen, Ming‐Hui Zhao

**Affiliations:** ^1^ Renal DivisionDepartment of MedicinePeking University First HospitalInstitute of NephrologyPeking UniversityKey Laboratory of Renal DiseaseMinistry of Health of ChinaBeijingChina

**Keywords:** HMGB1, ANCA, neutrophils, glomerular endothelial cells

## Abstract

Our previous studies demonstrated that high mobility group box‐1 (HMGB1), a typical damage‐associated molecular pattern (DAMP) protein, is associated with the disease activity of antineutrophil cytoplasmic antibody (ANCA)‐associated vasculitis (AAV). Moreover, HMGB1 participates in ANCA‐induced neutrophil activation. The current study aimed to investigate whether HMGB1 regulated the interaction between neutrophils and glomerular endothelial cells (GEnC) in the presence of ANCA. Correlation analysis on HMGB1 levels in AAV patients and soluble intercellular cell adhesion molecule‐1 (sICAM‐1) levels or vascular endothelial growth factor (VEGF) levels, which are markers of endothelial cell activation, was performed. The effect of HMGB1 on neutrophil migration towards GEnC, respiratory burst and degranulation of neutrophils in coculture conditions with GEnC was measured. The activation of neutrophils, the activation and injury of GEnC, and the consequent pathogenic role of injured GEnC were evaluated. Plasma levels of HMGB1 correlated with sICAM‐1 and VEGF (*r* = 0.73, *P* < 0.01; *r* = 0.41, *P* = 0.04) in AAV patients. HMGB1 increased neutrophil migration towards GEnC, as well as respiratory burst and degranulation of neutrophils in the presence of ANCA in the coculture system. In the presence of robust neutrophil activation, GEnC were further activated and injured in the coculture system of GEnC and neutrophils. In addition, injured GEnC could produce TF‐positive leuco‐endothelial microparticles and endothelin‐1 (ET‐1), while NF‐κB was phosphorylated (S529) in the injured GEnC. Plasma levels of HMGB1 correlated with endothelial cell activation in AAV patients. HMGB1 amplified neutrophil activation and the activation and injury of GEnC in the presence of ANCA.

## Introduction

Antineutrophil cytoplasmic antibody (ANCA)‐associated vasculitis (AAV) consists of granulomatosis with polyangiitis (GPA, previously named Wegener's granulomatosis), microscopic polyangiitis (MPA) and eosinophilic granulomatosis with polyangiitis (EGPA) 1. One of the hallmarks of AAV is massive endothelial injury, which involves, in particular, GEnC and results in necrotizing vasculitis. ANCAs, the serological markers for the aforementioned primary small vessel vasculitis, are involved in inducing and amplifying endothelial injury in AAV 2, 3, 4, 5, 6.

High mobility group box‐1 (HMGB1) is ubiquitously expressed within the nucleus, where it stabilizes the nucleosome structure and induces DNA bending 7. In response to specific stimuli, HMGB1 can be released from various cells to become a pro‐inflammatory mediator 8. In our recent studies, we found that circulating and urinary levels of HMGB1 closely correlate with disease activity and renal damage in AAV patients 9, 10.

Neutrophils are necessary for the pathogenesis of AAV 6. At the same time, endothelium–neutrophil interactions are also essential to allow the movement of neutrophils towards inflammatory sites and regulate neutrophil activation in AAV 11. We previously demonstrated that HMGB1 participates in ANCA‐induced neutrophil activation and neutrophil extracellular traps (NETs) formation, indicating a pathogenic role of HMGB1 in AAV 12, 13, 14. On the other hand, HMGB1 could also activate endothelial cells and increase their expression of intercellular adhesion molecules and release of pro‐inflammatory mediators 15, 16. Given the pro‐inflammatory effect of HMGB1 on both neutrophil and GEnC in the presence of ANCA, we suggested that HMGB1 could enhance endothelium–neutrophil interactions by amplifying neutrophil activation and the activation and injury of GEnC.

## Materials and methods

### Reagents

Recombinant HMGB1 protein was purchased from R&D Systems (C23‐C45 disulphide C106 thiol form) (Abingdon, UK). The endotoxin levels of HMGB1 were below the detection limit (0.125 EU/ml) of the Limulus assay (Sigma‐Aldrich, St Louis, MO, USA). Fluorescein isothiocyanate (FITC)‐labelled MAb against tissue factor (TF) (CJ4068) was obtained from American Diagnostica (Greenwich, CO, USA). Allophycocyanin (APC)‐labelled annexin V, phycoerythrin (PE)‐labelled CD31, APC‐labelled CD31 and isotype control mouse immunoglobulin G1 (IgG1) labelled with FITC, APC and PE were obtained from BioLegend (San Diego, CA, USA). One‐micrometre beads and reference beads were purchased from Sigma‐Aldrich (St Louis, MO, USA). PE‐labelled mouse anti‐NF‐κB p65 (pS529) was purchased from BD Biosciences (San Jose, CA, USA).

### Patients

Blood samples of 25 AAV patients with positive MPO‐ANCA at the initial onset and before commencing immunosuppressive therapy, who were diagnosed at Peking University First Hospital from August 2013 to June 2014, were collected in this study. All the patients met the Chapel Hill Consensus Conference (CHCC) definition of AAV and identified as MPA according to the European Medicines Agency (EMEA) algorithm 1, 17. Patients with secondary vasculitis, infections, traumas, cancers, other inflammatory diseases or comorbid renal diseases were excluded. Venous blood was collected into purple cap vacuum blood collection tubes with EDTA. Whole blood samples were centrifuged at 1000 × g for 10 min. at 4°C. Plasma was then obtained and stored at −80°C until use. The research was in compliance with the Declaration of Helsinki and approved by the clinical research ethics committee of the Peking University First Hospital.

### Measurement of plasma HMGB1

Plasma levels of HMGB1 were measured using commercially available ELISA kits (Shino‐TEST, Japan) according to the manufacturer's instructions.

### Measurement of plasma sICAM‐1 and VEGF

Levels of sICAM‐1, VEGF and soluble vascular cell adhesion molecule‐1 (sVCAM‐1), which are considered biomarkers of endothelial cell activation and injury 18, were tested using the BD™ cytometric bead array (CBA) Flex Set (BD Bioscience Pharmingen, San Diego, CA, USA). The assay was conducted according to the manufacturer's instructions, as previously described 19. Briefly, the CBA Flex Set contains various bead populations with distinct fluorescence intensity and the appropriate phycoerythrin (PE) detection reagent and standard. The bead populations were coated with capture antibodies specific to ICAM‐1 and VEGF. Then, the bead populations were incubated with recombinant standards or test samples to form sandwich complexes. After the addition of PE‐conjugated detection antibodies, the samples were incubated again and then analysed using a flow cytometer (Accuri C6). The results were generated in graphic and tabular format using the CBA analysis software (BD Bioscience Pharmingen).

### Preparation of IgG

Normal IgG and ANCA‐positive IgG were prepared from plasma of normal volunteers and patients with active PR3‐ANCA‐ or MPO‐ANCA‐positive primary small vessel vasculitis using a HiTrap Protein G column on an AKTA‐FPLC system (GE Biosciences, South San Francisco, CA, USA). None of the patients had dual positivity of PR3‐ANCA and MPO‐ANCA. Preparation of IgG was performed according to the methods described previously 20. We obtained written informed consent from all participants in our study.

### Neutrophil isolation

Neutrophils were isolated from heparinized venous blood of healthy donors by density gradient centrifugation on PolymorphPrep (Nycomed, Oslo, Norway). Then, neutrophils were washed in Hanks balanced salt solution without Ca^2+^/Mg^2+^ (HBSS−/−; Chemical Reagents, Beijing, China) and suspended in HBSS with Ca^2+^/Mg^2+^ (HBSS+/+; Chemical Reagents, Beijing, China) to a concentration of 1 × 10^6^ cells/ml and used for further analysis.

### Cell culture

Primary GEnC (ScienCell, San Diego, CA, USA) were cultured in endothelial cell (EC) basal medium (ScienCell) supplemented with 10% FBS, 1% penicillin/streptomycin and 1% endothelial cell growth factor in the formation of a confluent endothelial cells monolayer. Confluence of the endothelial monolayers was determined by continued monitoring of transendothelial electrical resistance (TEER) 21 which plateaued at 20–25 Ω on the third day after seeding. The flasks for cell subculture were bio‐coated with human plasma fibronectin (Millipore, Billerica, MA, USA) according to the manufacturer's recommendation. For the neutrophil migration assay, GEnC were grown on the lower chamber of Costar Transwell with 5‐μm porous filters (Coming, Acton, MA, USA), while for the permeability assay, GEnC were grown on the upper chamber of Costar Transwell with 5‐μm porous filters. For the synchronization of cell cycle, GEnC monolayers were starved in basal medium without serum and endothelial cell growth supplement for 12 hrs without bio‐coating. GEnC in selected wells were pre‐incubated for 4 hrs with 10 ng/ml HMGB1, which was comparable to the circulating HMGB1 levels in active AAV patients, as demonstrated in our previous study, while plasma levels of HMGB1 in normal controls were significantly lower and within the detection level 9. All experiments were performed using GEnC at passage number 3–5. All cells in culture and subsequent experiments were incubated at 37°C in 5% CO2.

### Neutrophil migration assay

For the migration assays, neutrophils were loaded with the fluorochrome 2,7‐bis‐(2‐carboxyethyl)‐5‐(and‐6)‐carboxyfluorescein, acetoxymethyl ester (BCECF‐AM; Life Technologies, Eugene, USA) according to the manufacturer's instructions. Briefly, neutrophils, which had been primed using HMGB1 at 10 ng/ml or buffer, were loaded with 2 μM BCECF for 15 min. at room temperature and then washed several times before being added into the upper chambers of Costar Transwell with 5‐μm porous filters. After 2 hrs, neutrophils that had migrated into the lower chamber were quantified by the fluorescent intensity (FI) of BCECF using a microplate fluorescence reader (TristarTM LB941) with filter settings of 485 nm (excitation) and 538 nm (emission). The migration of neutrophils was calculated according to the following formula:

Migration rate=FI (lower chamber)∗100%/(FI (upper chamber)+FI (lower chamber))

### Measurement of neutrophil activation in the coculture system of GEnC and neutrophils

#### Measurement of degranulation by lactoferrin quantification

Lactoferrin, an iron‐binding multifunctional glycoprotein that is an abundant component of the specific granules of neutrophils 22, was considered a biomarker of neutrophil degranulation 23. Neutrophils were primed with 10 ng/ml HMGB1 or buffer for 30 min. and then washed once with PBS and added onto GEnC monolayer in equal amounts, followed by stimulation with ANCA‐positive IgG, normal IgGs or buffer for 4 hrs. Supernatants were collected and used for ELISA analysis. Lactoferrin levels in the neutrophil supernatant were tested by ELISA using a commercial kit following the manufacturer's instructions (USCNK, Wuhan, China).

#### Measurement of respiratory burst by oxidation of 2,7‐dichlorodihydrofluorescein diacetate (DCFH‐DA)

2,7‐Dichlorodihydrofluorescein diacetate (DCFH‐DA, Sigma‐Aldrich) was used for ROS capture 24. DCFH‐DA is cleaved intracellularly by nonspecific esterases to form highly fluorescent 2,7‐dichlorofluorescein (DCF) upon oxidation by ROS. DCFH‐DA working solution was added directly to the medium to a final concentration of 10 μM and then incubated at 37°C for 15 min. 25. Neutrophils were primed with 10 ng/ml HMGB1 or buffer for 30 min. and then washed once with PBS and added onto GEnC monolayer in equal amounts, followed by stimulation with ANCA‐positive IgG, normal IgGs or buffer for 4 hrs. After stimulation, fluorescence was measured using a microplate fluorescence reader (TristarTM LB941) with filter settings of 485 nm (excitation) and 538 nm (emission).

According to a dose–response curve for HMGB1 in priming neutrophils (Supplementary Material and Supplementary Figure 1), the effect of HMGB1 on priming neutrophils, which was further in accordance with that of activating neutrophils, was roughly dose dependent.

### Measurements of GEnC activation and injury in the coculture system of GEnC and neutrophils

#### Measurement of sICAM‐1 and sVCAM‐1 in the supernatants

Levels of sICAM‐1 and sVCAM‐1 in the supernatants were tested using the BD™ CBA Flex Set as described above.

#### Permeability assay

GEnC monolayer permeability was determined using FITC‐labelled bovine serum albumin (Sigma‐Aldrich, St Louis, MO, USA) as previously described 26. Cells were grown on the upper chamber of Costar Transwell with 3‐μm porous filters until confluent. After coculture with neutrophils, the tracer protein FITC‐albumin was added to the upper chamber for 45 min. and samples were collected from both the upper and lower chambers for fluorometric analysis. Fluorescence was measured in a microplate fluorescence reader (TristarTM LB941) with filter settings of 485 nm (excitation) and 538 nm (emission). These readings were then used to determine the permeability coefficient of albumin, which is indicative of vascular barrier disruption. Data are presented as normalized to the controls for each test.

### Evaluation of the effect of injured GEnC on exacerbating inflammation and damage

#### Detection of tissue factor (TF)‐positive leuco‐endothelial microparticles (MPs)

Leuco‐endothelial MPs have been reported as tightly associated with endothelial dysfunction 27. Moreover, TF‐expressing MPs might promote hypercoagulability in AAV 28. Thus, TF‐positive leuco‐endothelial MPs isolated from the coculture system were here employed to reflect the effect of injured GEnC on exacerbating inflammation and damage. All buffers used in the isolation and detection of MPs were filtered through a 0.2‐mm filter. Supernatants obtained after stimulation were centrifuged at 200 × *g* for 5 min. to eliminate cells, then at 1500 × *g* for 15 min. to eliminate large debris and finally at 17,000 × *g* for 1 hr to pellet the MPs 29, 30. MPs were analysed by flow cytometry as previous described 28. One‐micrometre beads were used to set the correct gating for MPs, and reference beads were used to calculate the numbers of MPs in each stimulation group. Leuco‐endothelial MPs were stained with APC‐annexin V and PE‐CD31 in the presence of CaCl_2_ (5 mM) according to the recommendation of the supplier. FITC‐TF was used to determine TF expression on the isolated MPs.

#### Measurement of ET‐1

Endothelial ET‐1 is responsible for elevation of TGF‐β, which promotes the process of fibrosis 31. Thus, ET‐1 levels in the coculture system were also employed to quantify the effect of injured GEnC on exacerbating inflammation and damage. ET‐1 was measured in duplicate samples by ELISA (R&D Systems, Minneapolis, MN). ELISA was performed according to the instruction provided by the manufacturer.

### Analysis of NF‐κB phosphorylation (pS529) in injured GEnC

Cells in the coculture system of GEnC and neutrophils were digested using trypsin to maintain in suspension. After washing, suspended cells were stained with a saturating dose of APC‐CD31 for 15 min. Then, cells were fixed with BD Cytofix™ buffer for 10 min. at 37°C, permeabilized (BD™ Phosflow Perm Buffer III) on ice for 30 min. and stained with PE Mouse anti‐NF‐κB p65 (pS529). After washing with phosphate buffered saline (PBS), prepared cells were analysed using a flow cytometer (Accuri C6). Cells were gated in forward/sideward scatter (FSC/SSC) and data were collected from 10,000 cells per sample.

### Statistical analysis

Quantitative data were expressed as the means ± S.D. (for data that were normally distributed) or median and quartiles (for data that were not normally distributed), as appropriate. The normality of the data was evaluated using the kurtosis and skewness (both the absolute valves were less than 3). Differences in quantitative parameters between groups were assessed using one‐way anova analysis (for data that were normally distributed) or Kruskal–Wallis test (for data that were not normally distributed), as appropriate. Differences were considered significant when *P* < 0.05. Analysis was performed using the SPSS statistical ssoftware package (version 13.0, Chicago, IL, USA).

## Results

### The plasma levels of HMGB1 correlate with markers of endothelial cell activation in AAV patients

**Figure 1:**
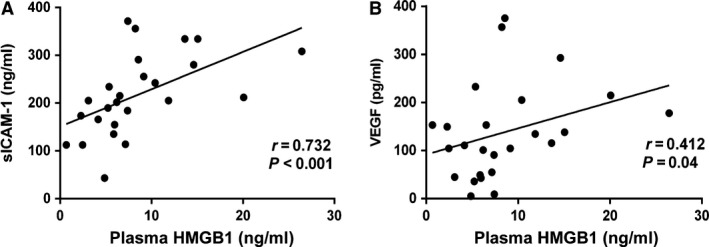
The plasma levels of HMGB1 correlate with the plasma levels sICAM‐1 and VEGF. The plasma levels of HMGB1 correlate with the plasma levels sICAM‐1 (**A**) and VEGF (**B**), which are markers of endothelial cell activation.

The levels of plasma HMGB1 were determined by ELISA, while sICAM‐1 and VEGF levels were measured using CBA Flex Set. Among the 25 patients with active AAV, plasma levels of HMGB1 correlated with plasma levels of soluble ICAM‐1 (*r* = 0.73, *P* < 0.01) and VEGF (*r* = 0.41, *P* = 0.04) (Fig. 1).

### HMGB1 increases neutrophil migration towards GEnC

**Figure 2:**
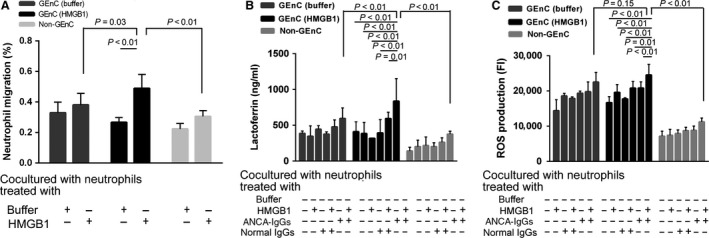
HMGB1 increases neutrophil migration towards GEnC, as well as respiratory burst and degranulation of neutrophils in the coculture system of GEnC and neutrophils. (**A**) HMGB1 increases neutrophil migration towards GEnC. (**B**) HMGB1 increases degranulation of neutrophils stimulated by HMGB1 plus ANCA‐positive IgGs. (**C**) HMGB1 increases respiratory burst of neutrophils stimulated by HMGB1 plus ANCA‐positive IgGs. Dark grey colour bars show untreated GEnC, black colour bars show HMGB1‐treated GEnC, and light grey colour bars show no‐GEnC‐grown. Bars represent the mean ± S.D. of repeated measurements of five independent experiments.

Compared with non‐treated GEnC or no‐GEnC‐grown groups, migration of HMGB1‐primed neutrophils towards HMGB1‐treated GEnC was significantly higher (49% ± 9% *versus* 38% ± 7%, *P* = 0.03; 49% ± 9% *versus* 30% ± 4%, *P* < 0.01, respectively). Compared with non‐primed neutrophils, migration towards GEnC of HMGB1‐primed neutrophils was significantly higher in HMGB1‐treated GEnC groups (49% ± 9% *versus* 27% ± 3%, *P* < 0.01) (Fig. 2A).

We also conducted MPO activity assay to quantify the cell amounts according to the methods described by Hu *et al*. 32. The results were in line with our previous results using BCECF probe (Supplementary Material 2). The viability rate of neutrophils was almost 90%~93% after incubating with/without HMGB1 or with/without BCECF for 3 hrs, indicating the intracellular pH might just experience a minimal change (Supplementary Figure 2).

We also measured the production of IL‐8, a well‐documented chemotactic factor on neutrophils, in the cocultured system (Supplementary Material and Supplementary Figure 3). It was found that IL‐8 levels were the highest in the supernatant of cocultured system in which neutrophils were primed by HMGB1 and GEnC were previously incubated with HMGB1. This was in line with the result of migration assay, indicating that HMGB1 could enhance the interaction between endothelium and neutrophils in ANCA‐associated vasculitis, to some degree, by promoting IL‐8 production.

### HMGB1 increases respiratory burst and degranulation of neutrophils in the coculture system of GEnC and neutrophils

We investigated whether HMGB1‐primed neutrophils for ANCA‐induced respiratory burst and degranulation in the coculture system of GEnC and neutrophils. ANCA‐IgGs were prepared from two patients with active PR3‐ANCA‐positive vasculitis, two patients with active MPO‐ANCA‐positive vasculitis and two healthy volunteers, respectively. Neutrophils from these six healthy donors were analysed. Degranulation was determined by measuring lactoferrin concentration in the supernatant of neutrophils stimulated by HMGB1 plus ANCA‐positive IgGs. Compared with unstimulated neutrophils, the concentration of lactoferrin increased significantly in the supernatant of HMGB1‐primed neutrophils induced by ANCA‐positive IgGs in the untreated GEnC groups, the HMGB1‐treated GEnC groups or the no‐GEnC‐grown groups (596 ± 145 ng/ml *versus* 387 ± 32 ng/ml, *P* < 0.01; 836 ± 315 ng/ml *versus* 411 ± 178 ng/ml, *P* < 0.01; 377 ± 40 ng/ml *versus* 141 ± 52 ng/ml, *P* < 0.01, respectively). No obvious degranulation was observed with HMGB1, ANCA‐IgG alone, normal IgG alone or HMGB1 plus normal IgG, in each group. Compared with the untreated GEnC groups or the no‐GEnC‐grown groups, neutrophils in the HMGB1‐treated GEnC groups had significantly more vigorous degranulation activity (836 ± 315 ng/ml *versus* 596 ± 145 ng/ml, *P* < 0.01; 836 ± 315 ng/ml *versus* 377 ± 40 ng/ml, *P* < 0.01, respectively) (Fig. 2B).

Respiratory burst was determined by measuring FI of the cocultured cells. Compared with non‐primed neutrophils, the FI value increased significantly in HMGB1‐primed neutrophils activated with ANCA‐positive IgGs in the untreated GEnC groups, HMGB1‐treated GEnC groups or no‐GEnC‐grown groups (22545 ± 2733 *versus* 14422 ± 3081, *P* < 0.01; 24532 ± 3042 *versus* 16700 ± 1694, *P* < 0.01; 11165 ± 766 *versus* 7173 ± 1406, *P* < 0.01, respectively). No obvious respiratory burst activity was observed in neutrophils incubated with HMGB1, ANCA‐IgG alone, normal IgG alone or HMGB1 plus normal IgG, in each group. Compared with the no‐GEnC‐grown groups, neutrophils in the GEnC‐grown groups had significantly more vigorous respiratory burst activity (22545 ± 2733 *versus* 11165 ± 766, *P* < 0.01; 24532 ± 3042 *versus* 11165 ± 766, *P* < 0.01, respectively), while there was no significant difference between the untreated GEnC groups and the HMGB1‐treated GEnC groups (*P* = 0.15) (Fig. 2C).

### Under the condition of vigorous activation of neutrophils, GEnC are further activated and injured in the coculture system of GEnC and neutrophils

In the HMGB1‐treated GEnC groups, we next studied the effect of HMGB1 on the activation and subsequent injury of GEnC.

**Figure 3:**
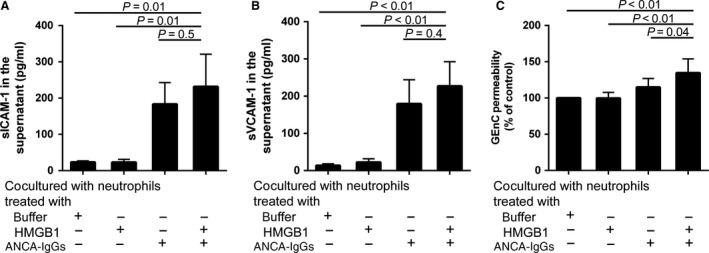
Under the condition of vigorous activation of neutrophils, GEnC are further activated and injured in the coculture system of GEnC and neutrophils. (**A–B**) The levels of both sICAM‐1 and sVCAM‐1 increase significantly in the supernatants of GEnC cocultured with HMGB1‐primed neutrophils activated with patients‐derived ANCA‐positive IgGs. (**C**) The levels of vascular barrier disruption increase significantly in GEnC cocultured with HMGB1‐primed neutrophils activated with patients‐derived ANCA‐positive IgGs. Bars represent the mean ± S.D. of repeated measurements of five independent experiments.

sICAM‐1 and sVCAM‐1 were considered as markers of endothelial cell activation. Compared with GEnC cocultured with non‐primed neutrophils, the levels of both sICAM‐1 and sVCAM‐1 increased significantly in the supernatants of GEnC cocultured with HMGB1‐primed neutrophils activated with patients‐derived ANCA‐positive IgGs (232 ± 89 pg/ml *versus* 23 ± 4 pg/ml, *P* = 0.01; 227 ± 65 pg/ml *versus* 14 ± 4 pg/ml, *P* < 0.01, respectively). However, compared with GEnC cocultured with non‐primed neutrophils, levels of these markers also significantly higher in the supernatants of GEnC cocultured with non‐primed neutrophils activated with patients‐derived ANCA‐positive IgGs alone (184 ± 59 pg/ml *versus* 23 ± 4 pg/ml, *P* = 0.01; 179 ± 65 pg/ml *versus* 14 ± 4 pg/ml, *P* < 0.01, respectively), suggesting a predominant role of ANCA on activating GEnC. There was no significant difference between these cells and GEnC cocultured with HMGB1‐primed neutrophils activated with patients‐derived ANCA‐positive IgGs (*P* = 0.5; *P* = 0.4, respectively) (Fig. 3A and B).

Endothelial cell permeability assay was performed to study the vascular barrier disruption in different experimental groups. Compared with GEnC cocultured with non‐primed neutrophils, GEnC cocultured with HMGB1‐primed neutrophils and GEnC cocultured with non‐primed neutrophils activated with ANCA‐positive IgGs, the levels of vascular barrier disruption increased significantly in GEnC cocultured with HMGB1‐primed neutrophils activated with patients‐derived ANCA‐positive IgGs (135% ± 19% *versus* 100%, *P* < 0.01; 135% ± 19% *versus* 100% ± 8%, *P* < 0.01; 135% ± 19% *versus* 117% ± 15%, *P* = 0.04, respectively. Data are shown as percentage of control (Fig. 3C).

### Under the condition of vigorous activation of neutrophils, injured GEnC participate in the production of TF‐positive leuco‐endothelial MPs and ET‐1

Next, we evaluated the pro‐inflammatory effect of injured GEnC in the HMGB1‐treated GEnC groups.

**Figure 4:**
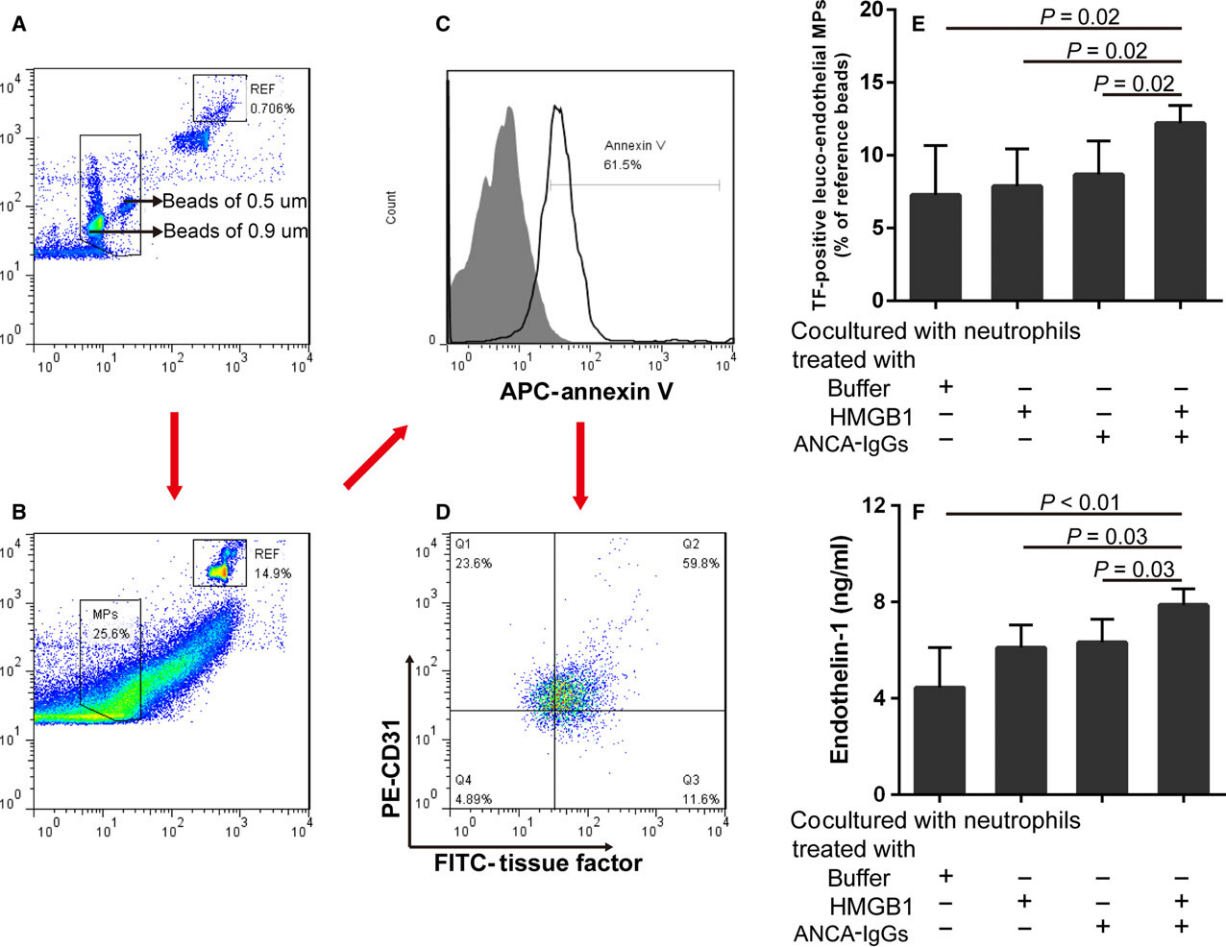
Under the condition of vigorous activation of neutrophils, injured GEnC participate to the production of TF‐positive leuco‐endothelial MPs and ET‐1. (**A–D**) Gating strategy of TF‐positive leuco‐endothelial MPs. (**E**) The levels of TF‐positive leuco‐endothelial MPs increase significantly in GEnC cocultured with HMGB1‐primed neutrophils activated with patients‐derived ANCA‐positive IgGs. (**F**) The levels of ET‐1 increase significantly in GEnC cocultured with HMGB1‐primed neutrophils activated with patients‐derived ANCA‐positive IgGs. Bars represent the mean ± S.D. of repeated measurements of five independent experiments.

TF‐positive leuco‐endothelial MPs were gated as shown in Figure 4A–D. Compared with GEnC cocultured with non‐primed neutrophils, GEnC cocultured with HMGB1‐primed neutrophils and GEnC cocultured with non‐primed neutrophils activated with ANCA‐positive IgGs, the levels of TF‐positive leuco‐endothelial MPs increased significantly in GEnC cocultured with HMGB1‐primed neutrophils activated with patients‐derived ANCA‐positive IgGs (12 ± 1 *versus* 7 ± 3, *P* = 0.02; 12 ± 1 *versus* 8 ± 3, *P* = 0.02; 2 ± 1 *versus* 9 ± 2, *P* = 0.02, respectively). Data were shown as normalized to reference beads (Fig. 4E).

Compared with GEnC cocultured with non‐primed neutrophils, GEnC cocultured with HMGB1‐primed neutrophils and GEnC cocultured with non‐primed neutrophils activated with ANCA‐positive IgGs, the levels of ET‐1 increased significantly in GEnC cocultured with HMGB1‐primed neutrophils activated with patients‐derived ANCA‐positive IgGs (7.9 ± 0.7 ng/ml *versus* 4.4 ± 1.7 ng/ml, *P* < 0.01; 7.9 ± 0.7 ng/ml *versus* 6.1 ± 1.1 ng/ml, *P* = 0.03; 7.9 ± 0.7 ng/ml *versus* 6.3 ± 0.9 ng/ml, *P* = 0.03, respectively) (Fig. 4F).

### Under the condition of vigorous activation of neutrophils, NF‐κB is phosphorylated (S529) in the injured GEnC

**Figure 5:**
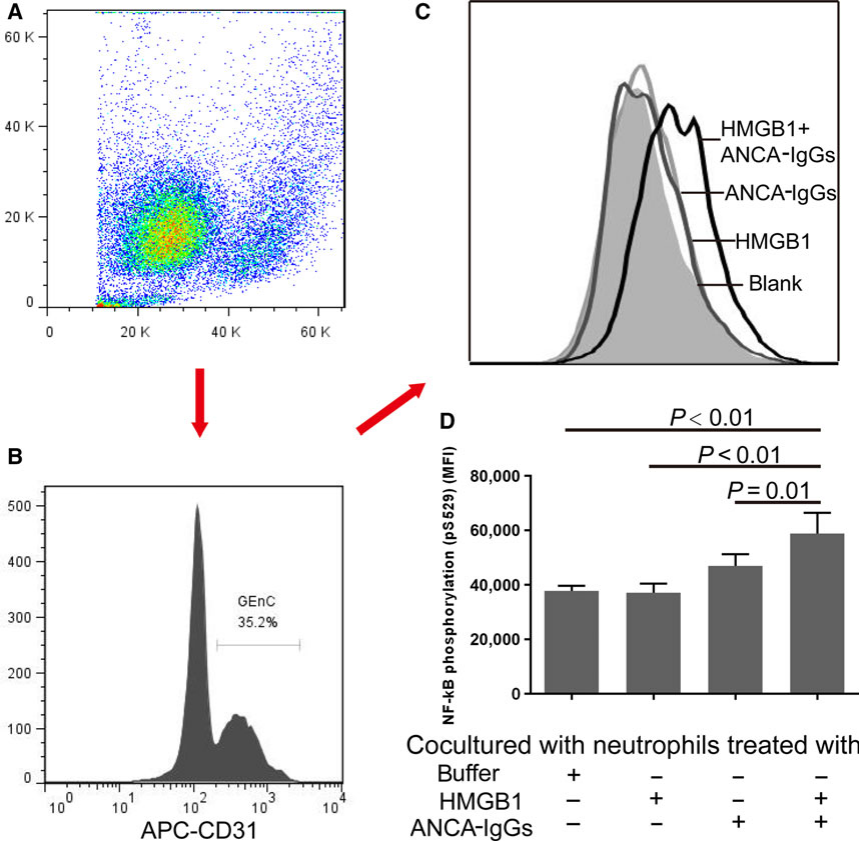
Under the condition of vigorous activation of neutrophils, NF‐κB is phosphorylated (S529) in the injured GEnC. (**A–B**) Gating strategy of GEnC in the coculture system of GEnC and neutrophils. (**C**) A representative histogram of NF‐κB phosphorylation (pS529) in GEnC. (**D**) The levels of NF‐kB phosphorylation (pS529) increase significantly in GEnC cocultured with HMGB1‐primed neutrophils activated with patients‐derived ANCA‐positive IgGs. Bars represent the mean ± S.D. of repeated measurements of three independent experiments.

Compared with GEnC cocultured with non‐primed neutrophils, GEnC cocultured with HMGB1‐primed neutrophils and GEnC cocultured with non‐primed neutrophils activated ANCA‐positive IgGs, the levels of phosphorylation of NF‐κB increased significantly in GEnC cocultured with HMGB1‐primed neutrophils activated with patients‐derived ANCA‐positive IgGs (58857 ± 7613 *versus* 37861 ± 1746, *P* < 0.01; 58857 ± 7613 *versus*. 37152 ± 3335 ng/ml, *P <* 0.01; 58857 ± 7613 *versus* 47007 ± 4221, *P* = 0.01, respectively) (Fig. 5D). These results suggested that NF‐κB is phosphorylated (S529) in the injured GEnC.

## Discussion

In the current study, we observed that the plasma levels of HMGB1 correlated with markers of endothelial cell activation in patients with active AAV, indicating that HMGB1 is associated with endothelial cell injury in AAV. We showed that HMGB1 increased the migration of neutrophils towards GEnC‐ and ANCA‐induced neutrophil activation in a coculture system with GEnC. Then, under the condition of vigorous activation of neutrophils, GEnC were further activated, injured and contributed to exacerbating inflammation and damage.

Endothelium–neutrophil interactions are essential to regulate neutrophil activation in AAV 11. In particular, plasma proteins and oxidants, including serum albumin, endogenous ceramide and α1‐antitrypsin, prevent untimely intravascular activation of neutrophils in circulation 33, 34, 35, 36. Only in the presence of firm adhesion between neutrophils and ICAM‐1‐expressing endothelial cells, neutrophil responses can persist and result in degranulation and oxidative burst in the presence of ANCA 37, 38. Consistently, in our study, the neutrophils in the coculture system would undergo more vigorous activation than the neutrophils cultured alone. Moreover, this activation would be more severe if GEnC were pre‐treated with HMGB1. This response might be attributed to the effect of HMGB1 on the up‐regulation of ICAM‐1 expression on endothelial cells 15, 16.

Notably, there was no significant difference in GEnC activation between GEnC cocultured with HMGB1‐primed neutrophils activated with patients‐derived ANCA‐positive IgGs and GEnC cocultured with non‐primed neutrophils activated with patients‐derived ANCA‐positive IgGs. Previous reports suggested that ANCA plays a predominant role on activating GEnC. However, GEnC in our system were also pre‐treated with HMGB1. Nagao *et al*. reported a direct activation of mouse GEnC by antimoesin activity of anti‐MPO antibody. They identified a cross‐reactive molecule, which could be recognized by anti‐MPO antibody, existing on mouse GEnC. Later, the molecule was confirmed as moesin by mass spectrometry 5. On the other hand, Lee *et al*. 39 demonstrated that the HMGB1‐RAGE‐moesin axis could elicit severe inflammatory responses on human umbilical vein endothelial cells (HUVEC), during which HMGB1 exhibited an increase in phosphorylation of moesin and further secretion of moesin. Consistently, we found that HMGB1 enhances the direct ability of MPO‐ANCA to cause GEnC activation by increasing moesin expression to some degree (unpublished data), which might partially explain these controversial observations.

In the current study, we used ET‐1 to measure the pro‐inflammatory effect of injured GEnC on exacerbating inflammation and damage. Because neutrophils cannot release ET‐1, the ET‐1 detected in the system originated from GEnC. Using mice with a specific deletion of the ET‐1 gene in vascular endothelial cells, it was shown that endothelial ET‐1 was necessary not only to the elevation of TGF‐β but also to the increase in protein kinase C δ abundance and ERK1/2 activation, which result in chronic fibrosis 40, 41. In addition, Zager *et al*. 42 demonstrated that ET‐1 plays a critical role in post‐ischaemic acute kidney injury (AKI) progression to chronic kidney disease (CKD), indicating a role of ET‐1 in the chronicity of inflammation. Therefore, by exacerbating inflammation and damage, endothelial cells might change from victims into assaulters.

Due to the diverse effects of HMGB1 on neutrophils, endothelial cells, as well as lymphocytes, the complement system and the coagulation system 13, 43, 44, 45, 46, HMGB1 might be further involved in the pathogenesis of AAV. Thus, targeting HMGB1 might be an attractive therapeutic modality for inflammatory vascular diseases. There are many studies showing a promising protective effect by blocking HMGB1 in experimental models of several inflammatory diseases, including endotoxemia, arthritis and ischaemia–reperfusion injury 47, 48, 49, 50, 51. Combined all above findings, an anti‐HMGB1‐based therapeutic strategy might also be useful in AAV, which needs further studies to confirm.

In conclusion, HMGB1 aggravates the activation of neutrophils and the activation and injury of GEnC in the presence of ANCA in a coculture system of neutrophils and GEnC. The current findings help us understand the exact pathogenic role of ANCA in AAV, thus providing potential clues for intervention strategies.

## Funding

This study is supported by three grants of the National Natural Science Fund (No. 81425008, No. 81321064 and No. 81300599) and the grant from National Key Research and Development Program (No. 2016YFC0906102).

## Conflict of interest

This manuscript has been read and approved by all authors. It has not been published elsewhere. No conflict of interest was declared.

## Supporting information


**Figure S1.** Dose‐response curves for HMGB1 in priming neutrophils.

**Figure S2.** The viability rate of neutrophils after incubating with/without HMGB1 or with/without BCECF.

**Figure S3.** The production of IL‐8 in the co‐cultured system.
