# Supplementary figures and images for: HMGB1 contributes to glomerular endothelial cell injury in ANCA‐associated vasculitis through enhancing endothelium–neutrophil interactions
Source: J Cell Mol Med. 2017 Feb 9;21(7):1351–60. doi: 10.1111/jcmm.13065 (PMC5487910; doi:10.1111/jcmm.13065)

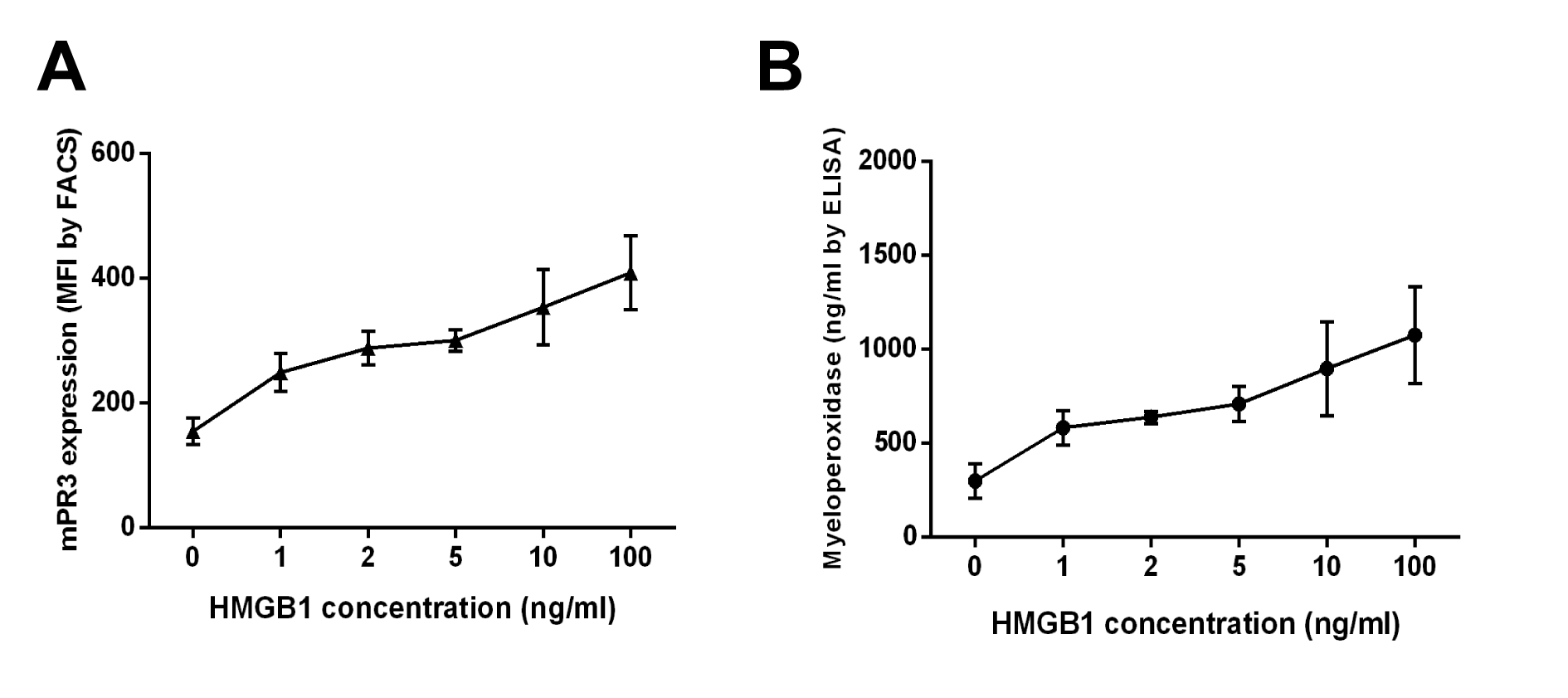

Supplement: Supplementary file 1 — Figure S1. Dose‐response curves for HMGB1 in priming neutrophils. [file JCMM-21-1351-s001.tif]

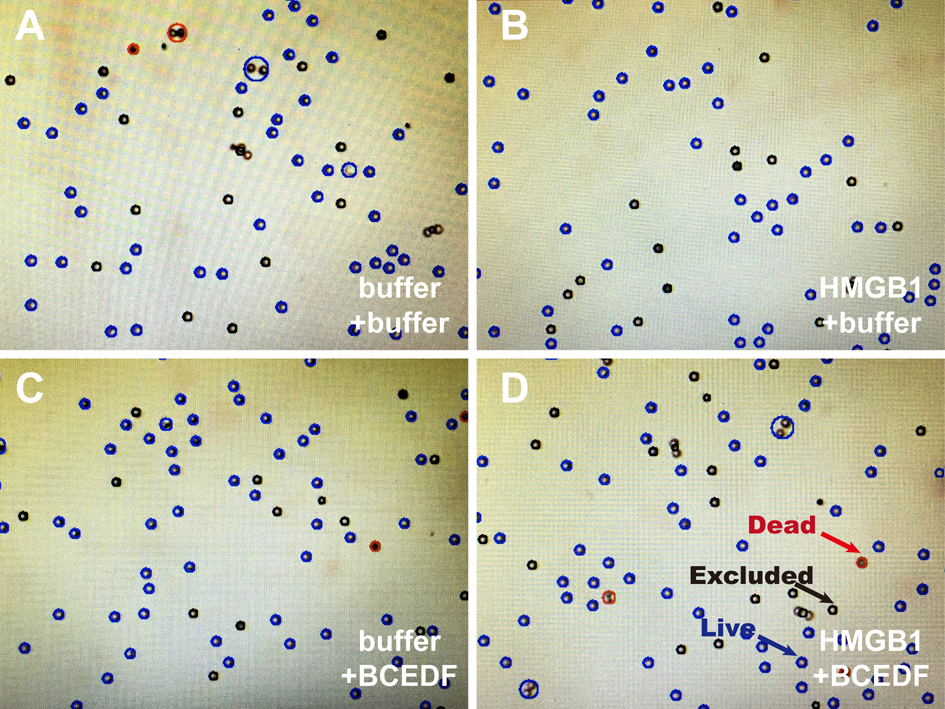

Supplement: Supplementary file 2 — Figure S2. The viability rate of neutrophils after incubating with/without HMGB1 or with/without BCECF. [file JCMM-21-1351-s002.tif]

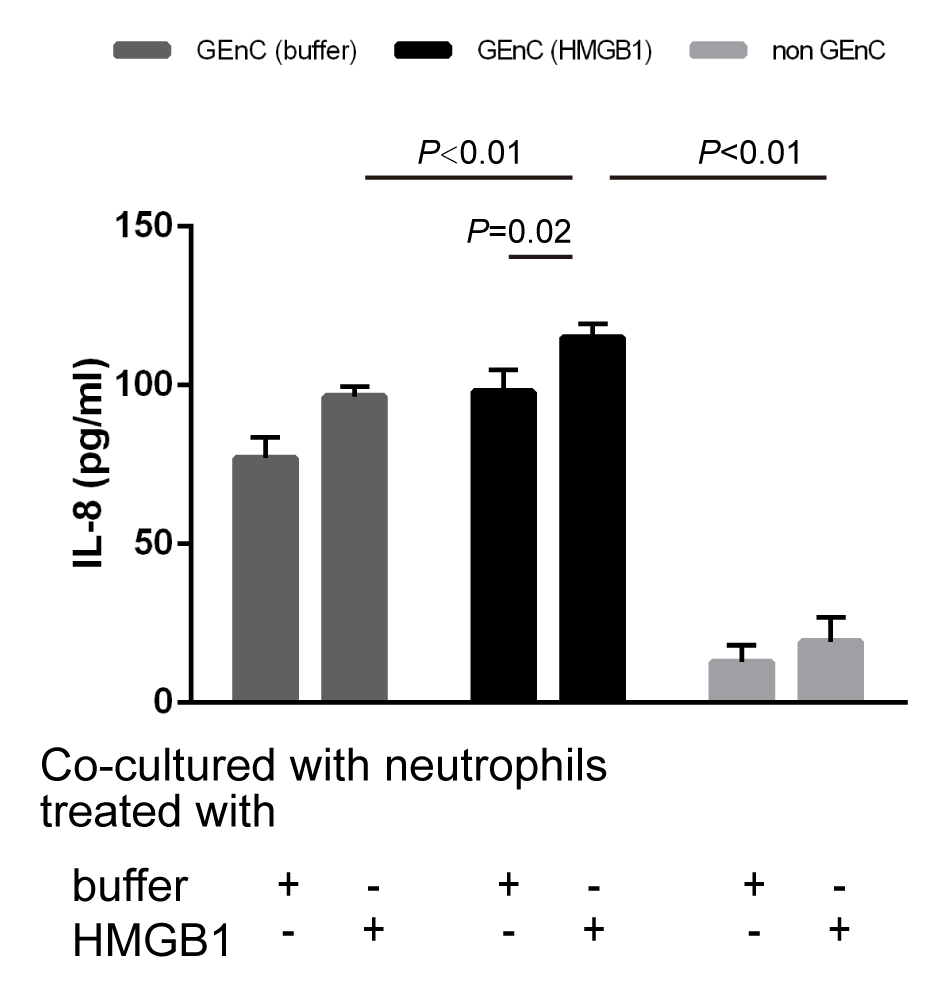

Supplement: Supplementary file 3 — Figure S3. The production of IL‐8 in the co‐cultured system. [file JCMM-21-1351-s003.tif]
