# Supplementary material for: HMGB1 contributes to glomerular endothelial cell injury in ANCA‐associated vasculitis through enhancing endothelium–neutrophil interactions
Source: J Cell Mol Med. 2017 Feb 9;21(7):1351–60. doi: 10.1111/jcmm.13065 (PMC5487910; doi:10.1111/jcmm.13065)
Supplement: Supplementary file 4 [file JCMM-21-1351-s004.doc]

**Supplementary Material**

**Supplementary material-1**

**Dose-response curves for HMGB1 in priming neutrophils**

**Materials and Methods**

**Neutrophil isolation**

Fresh blood of healthy donors was collected by three kinds of tubes containing different anticoagulant, i.e., sodium citrate, EDTA and heparin, respectively. The later procedure was as described in the main text of the manuscript.

**Membrane expression of PR3 (mPR3) on neutrophils after priming**

Flow cytometry was used to evaluate mPR3 expression on neutrophils. All further steps were performed on ice and washing steps were carried out using HBSS +/+ containing 1% BSA. TruStain fcX (BioLegend) was used in all samples prior to the addition of antibodies to block nonspecific antibodies binding. Next, cells were stained with a saturating dose of FITC-conjugated mouse monoclonal IgG1 antibody directed against human PR3 (Abcam, Cambridge, UK) or with an irrelevant IgG1 control antibody for 30 min. Fluorescence intensity of FITC was analyzed using flow cytometry assessment of ANCA-antigen expression. Samples were analyzed using a FACScan (Becton Dickinson, Germany). Neutrophils were identified in the scatter diagram, and data were collected from 10,000 cells per sample. The level of PR3-expression was calculated as MFI of specific binding of the isotype control antibody.

**Detection of MPO in the supernatant of HMGB1-primed neutrophils by ELISA**

MPO in the supernatant of HMGB1-primed neutrophils was tested by ELISA using a commercial kit (USCNK, Wuhan, China). Cells were incubated with HMGB1 for 30 min at 37°C. Supernatant fluids were collected and used for ELISA analysis.

**Result**

The levels of mPR3 expression on neutrophils and MPO in the supernatant of neutrophils were roughly dose-dependent (**Supplementary Figure 1**).

**Supplementary material 2**

**Neutrophils migration by MPO activity assay**

**Materials and Methods**

**Neutrophil isolation**

Fresh blood of healthy donors was collected by three kinds of tubes containing different anticoagulant, i.e., sodium citrate, EDTA and heparin, respectively. The later procedure was as described in the main-text of the manuscript.

**Measurement of neutrophil migration**

Neutrophils were added into the upper chambers of Costar Transwell 5-μm porous filters without the probe. After 2h, neutrophils that migrated into the lower chamber were quantified by the MPO activity assay according to the methods described by Hu et al . Briefly, cells were lysed with 0.5% Triton-X-100 at 4°C for 20 minutes. The samples were transferred into wells of a flat-bottomed 96-well plate. Then 30% H_2_O_2_ and 0.4 g/L o-Phenylenediamine (Sigma, St Louis, MO, USA) in PBS was loaded in an equal volume as substrate for MPO. After the reaction, OD_450_ was measured using a plate reader.

The migration of neutrophils was calculated according to the following formula:

Migration rate = OD_450_ (lower chamber)*100%

/ (OD_450_ (upper chamber) + OD_450_ (lower chamber))

**Statistical analysis**

Quantitative data were expressed as the means±SD. Differences in quantitative parameters between groups were assessed using one-way ANOVA analysis (for the data were normally distributed). Differences were considered significant when P<0.05. Analysis was performed using the SPSS statistical software package (version 13.0, Chicago, USA).

**Result**

Compared with non-treated GEnC or no GEnC grown groups, migration of HMGB1-primed neutrophils towards HMGB1-treated GEnC was significantly higher (45%±3% vs. 30%±4%, *P*<0.01; 45%±3% vs. 25%±4%, *P*<0.01, respectively). Compared with non-primed neutrophils, migration towards GEnC of HMGB1-primed neutrophils was significantly increased in HMGB1-treated GEnC groups (45%±3% vs. 23%±2%, *P*<0.01). The results were in line with results using BCECF probe.

**References**

1**. Hu N, Westra J, Rutgers A, et a**l. Decreased CXCR1 and CXCR2 expression on neutrophils in anti-neutrophil cytoplasmic autoantibody-associated vasculitides potentially increases neutrophil adhesion and impairs migration*. Arthritis Res Th*er. 2011; 13: R201.

**Supplementary material 3**

**Measurement of IL-8 by endothelial cells in the co-cultured system**

**Materials and Methods**

**Neutrophil isolation**

Fresh blood of healthy donors was collected by three kinds of tubes containing different anticoagulant, i.e., sodium citrate, EDTA and heparin, respectively. The later procedure was as described in the main text of the manuscript.

**Glomerular endothelial cells culture**

Primary GEnC (ScienCell, San Diego, USA) were cultured in endothelial cell (EC) basal medium (ScienCell, San Diego, USA) supplemented with 10% FBS, 1% penicillin/streptomycin, and 1% endothelial cell growth factor in the formation of a confluent endothelial cells monolayer on the lower chamber of Costar Transwell with 5-μm porous filters (Coming, Acton, USA).

**Measurement of neutrophil migration**

Neutrophils were added into the upper chambers of Costar Transwell 5-μm porous filters without the probe. After 2h, neutrophils migrated into the lower chamber and the supernatant was collected.

**Measurement of IL-8 in the supernatant of co-cultured system**

IL-8 in the supernatant of co-cultured system was measured by ELISA (Dakewei, Beijing, China). ELISA was performed according to the instruction provided by the manufacturer.

**Statistical analysis**

Quantitative data were expressed as the means±SD. Differences in quantitative parameters between groups were assessed using one-way ANOVA analysis (for the data were normally distributed). Differences were considered significant when *P*<0.05. Analysis was performed using the SPSS statistical software package (version 13.0, Chicago, USA).

**Result**

Compared with non-treated GEnC or no GEnC grown groups, the levels of IL-8 in the supernatant of co-cultured system were significantly higher (115.1±4.4pg/ml vs. 96.4±3.2pg/ml, P<0.01; 115.1±4.4pg/ml vs. 19.2±7.8pg/ml, P<0.01, respectively). Further in HMGB1-treated GEnC groups, compared with that of the co-cultured system in which neutrophils were not primed, the levels of IL-8 in the supernatant of co-cultured system in which neutrophils were primed by HMGB1 were significantly higher (115.1±4.4pg/ml vs. 97.2±7.1pg/ml, *P*=0.02) (**Supplementary Figure 3**).

**Legends for Supplementary Figures**

**Supplementary Figure 1. Dose-response curves for HMGB1 in priming neutrophils**

The levels of mPR3 expression on neutrophils (A) and MPO in the supernatant of neutrophils (B) were roughly dose-dependent.

Bars represent mean±SD of repeated measurements of 4 independent experiments.

**Supplementary Figure 2. The viability rate of neutrophils after incubating with/without HMGB1 or with/without BCECF**

Representative images of neutrophils after incubating without HMGB1 or BCECF (A), with HMGB1 but without BCECF (B), without HMGB1 but with BCECF (C) and with HMGB1 and BCECF (D).

**Supplementary Figure 3. The production of IL-8 in the co-cultured system**

The levels of IL-8 in the supernatant of the co-cultured systems with different stimuli.

Bars represent mean±SD of repeated measurements of 4 independent experiments.
